# COVID-19 and Serious Bacterial Infection in Febrile Infants Less Than 60 Days Old

**DOI:** 10.5811/westjem.2022.6.54863

**Published:** 2022-08-10

**Authors:** David Guernsey, Matthew Pfeffer, James Kimpo, Hector Vazquez, Jessica Zerzan

**Affiliations:** Maimonides Medical Center, Department of Emergency Medicine, Brooklyn, New York

## Abstract

**Introduction:**

The pandemic caused by severe acute respiratory syndrome coronavirus 2 (SARS-CoV-2) led to the coronavirus disease 2019 (COVID-19) pandemic that drastically impacted the United States. The evidence was not clear on how SARS-CoV-2 infection impacted children, given the high prevalence of SAR-CoV-2 infection. Febrile infants less than 60 days old are an ongoing challenge to risk-stratify for serious bacterial infection (SBI), including urinary tract infection (UTI), bacteremia, and meningitis. We hypothesized there would be a lower rate of SBI in SARS-CoV-2 positive febrile infants compared to those SARS-CoV-2 negative.

**Methods:**

This was a retrospective chart review with a nested, age-matched, case-control study performed from March 2020–June 2021. Infants less than 60 days old presenting with fever were assigned groups based on SARS-CoV-2 infection. Blood, urine, and cerebrospinal fluid cultures were used as the gold standard to diagnose SBI. We compared overall rate of SBI as well as individual rates of SBI between each group. We performed a subgroup analysis evaluating the age group 29–60 days old.

**Results:**

A total of 164 subjects met criteria for analysis: 30 COVID-19 positive and 134 COVID-19 negative subjects. Rate of SBI was 17.9% (95% confidence interval [CI]: 11.8–25.5%) in the COVID-19 negative group compared to 0% (95% CI: 0.0%-11.1%) in the COVID-19 group, which demonstrated statistical significance (p = 0.008). In the age-matched data, we found statistical significance for any SBI (p = <0.001). For individual rates of SBI, we found statistical significance for UTI (p = <0.001) and bacteremia (p = <0.001). The 29–60 days-old subgroup analysis did not achieve statistical significance (p = 0.11).

**Conclusion:**

This study demonstrated the utility of including SARS-CoV-2 infection as part of the risk stratification of febrile infants less than 60 days old. While overall there is a low incidence of bacteremia and meningitis in this age group, these results can contribute to existing literature and potentially help decrease invasive testing and exposure to broad-spectrum antibiotics.

## INTRODUCTION

In early March 2020, severe acute respiratory syndrome coronavirus 2 (SARS-CoV-2), a novel single-stranded RNA virus, led to a pandemic of coronavirus disease 2019 (COVID-19).^1^ Maimonides Medical Center (MMC) is an urban, community-based, academic hospital located in Brooklyn, NY, with the only children’s hospital in the largest borough of the largest city in the United States. At one point it was one of the epicenters of the COVID-19 pandemic. The pediatric emergency department (PED) is nested within the main ED staffed by pediatric emergency medicine-trained physicians with approximately 35,000 visits annually prior to the COVID-19 pandemic. The effect COVID-19 had on infants varied on presentation, symptoms, and severity of illness.^2^–^4^

Infants less than 60 days old presenting to the PED with fever are some of the most vulnerable patients due to their risk for serious bacterial infections (SBI), which include urinary tract infections (UTI), bacteremia, and bacterial meningitis. The reported rates of SBI for infants less than 60 days old ranges from 7.0–12.5% for low-risk infants to as high as 20% for high-risk infants.^5^,^6^ Clinicians are tasked with identifying those neonates who are high risk given the need for advanced testing and management in these patients. In 2019, the Febrile Infant Working Group of the Pediatric Emergency Care Applied Research Network (PECARN) published new criteria to help identify infants at low risk for SBI. Specifically, PECARN helped to risk-stratify infants 29–60 days old.^7^

Recently, the American Academy of Pediatrics (AAP) updated its guidelines for the evaluation and management of febrile infants 8–60 days old to further clarify risk stratification, creating new age-group definitions.^8^ This literature speaks to the ongoing evolution and discussion of the best approach to this patient population. Clinical prediction rules such as the AAP guidelines help guide patient management and disposition.^9^ Multiple studies have shown that febrile infants with viral infection such as respiratory syncytial virus (RSV) or influenza are at a lower risk of SBI when compared to those infants without viral infection.^10^,^11^ Recently published data evaluating the effect of SARS-CoV-2 infection on the risk for SBI has yielded similar results.^12^

The emergence of a new infectious disease presents challenges in the risk assessment of any patient, but particularly in this age group. Our goal in this paper was to identify the rate of SBI in febrile infants less than 60 days old presenting to the ED who were COVID-19 positive and then compare it to the rate of SBI in febrile infants less than 60 days old who were COVID-19 negative. We hypothesized that infection with SARS-CoV-2 in febrile infants ≤60 days-old would be associated with a lower risk of SBI than febrile infants ≤60 days old without SARS-CoV-2 infection.

## METHODS

This was a retrospective cohort study of febrile infants ages 60 days old or less who presented to the PED. Fever was considered any rectal temperature of 38°C (100.4°F) or greater in the prior 24 hours either at home or during the PED visit. The study period encompassed March 2020–June 2021. We identified patients by querying the electronic health record (EHR) for all infants ≤60 days old who presented to the PED during the defined study period. We reviewed each variable and how to extract the information from the subject’s chart. Patients were excluded if they were afebrile at home and during the PED visit, had received antibiotics within 48 hours of arrival to the PED, had a recent diagnosis of SBI, gestation age less than 35 weeks, or comorbidities such as congenital heart disease, chronic lung disease, or ventriculoperitoneal shunts, which place those patients at higher risk of complicated illness.

**Population Health Research Capsule**

What do we already know about this issue?

*The impact of COVID-19 on children has been less severe compared to adults, but the impact on high-risk, febrile neonates has not been clearly documented*.

What was the research question?

*What impact did concurrent COVID-19 infection on febrile neonates less than 60 days old have on serious bacterial infections?*

What was the major finding of the study?

*Rate of SBI for concurrent COVID-19 infection was 0% (95% CI 0–11%), statistically significantly lower (p = 0.008) than 18% (12–26%) found in COVID-19 negative patients*.

How does this improve population health?

*Febrile neonates are at high risk for serious bacterial infection. COVID 19 positive neonates may have a lower incidence of SBI, and allow for improved risk stratification and management*.

The PECARN and AAP guidelines vary in their recommendations on the evaluation and management of these infants, including laboratory studies, treatment, and disposition from the PED.^7^,^8^ This variation in recommendations and guidelines has led to inconsistent practice by physicians. As this study was a retrospective chart review, it had no impact on the physician’s evaluation and management decisions. Thus, we excluded those patients whose evaluation did not include a SARS-CoV-2 test and at least one bacterial culture (urine, blood, and/or cerebrospinal fluid [CSF]). Infants were classified based on their COVID-19 status as determined by real-time reverse transcription–polymerase chain reaction (RT-PCR) testing of nasopharyngeal specimens, performed using one of several platforms including BioFire Respiratory 2.1 Panel (BioFire Diagnostics LLC, Salt Lake City, UT), Respiratory Pathogen Panel 2 (GenMark Diagnostics, Inc, Carlsbad, CA), and Xpert Xpress SARS-CoV-2/Flu/RSV (Cepheid, Sunnyvale, CA). The decision to test for SARS-CoV-2 was at the discretion of the treating physician; however, all hospitalized neonates required COVID-19 testing prior to bed assignment.

Data was collected directly from the EHR and stored in Microsoft Excel (Microsoft Corp, Redmond, WA). Data collected included demographics, chief complaint, preceding signs and symptoms, maximum temperature, ED disposition, *International Classification of Diseases, 10^th^ Revision*, code diagnoses, length of stay if admitted to the hospital, and antibiotic therapy if treated with at least one dose. Laboratory data collected included white blood cell count, absolute neutrophil count, procalcitonin, urinalysis, urine culture, blood culture, CSF culture, respiratory viral panel (RVP), SARS-CoV-2 result, and chest radiograph result. The primary outcome was the presence of SBI, specifically urinary tract infection (UTI), bacteremia, or bacterial meningitis. A UTI was defined as a urine culture with at least 50,000 colony-forming units per milliliter of a single pathogen obtained via sterile catheterization. Bacteremia and bacterial meningitis were defined as growth of a single pathogen on either blood or CSF culture. We did not consider enteritis as part of SBI due to the low incidence at our institution and the lack of routine stool cultures.

Subjects were assigned to their respective group based on SARS-CoV-2 result. We analyzed the rate of overall SBI between COVID-19 positive and COVID-19 negative patients. A subgroup analysis was also performed examining the rate of each SBI (UTI, bacteremia, and meningitis) and the rate of invasive bacterial infections (IBI), which is defined as bacteremia and bacterial meningitis. We performed a nested, age-matched (+/− 2 days) case-control analysis on the data. An age-specific group of 29–60 days old analysis was completed comparing the rate of SBI between COVID-positive and negative groups. Finally, we completed an analysis in which subjects were assigned to viral infection positive or viral infection negative groups based on RVP results. The rate of any SBI, each individual SBI (UTI, bacteremia, and meningitis), and IBI were compared between the two groups.

We summarized all continuous variables with medians and interquartile ranges, and categorical variables were summarized with frequency and count. We used the Mann-Whitney U test to compare continuous variables. Chi-square or Fisher’s exact test was used when comparing categorical variables. For the matched data analysis, we performed McNemar’s test. All statistical analysis was performed using SPSS statistical software (IBM Corporation. Armonk, NY) Alpha was set at 0.05. This study was approved under expedited review by the institutional review board at MMC.

## RESULTS

We pulled 591 charts from the EHR that met age criteria. A total of 174 subjects met fever criteria for data collection. Of those, 10 subjects were removed because of absence of a SARS-CoV-2 result. No subject was excluded due to having at least one bacterial culture. All infants had urine and blood collected, while 49 (29.9%) of the patients did not have CSF collected. A complete RVP was not always readily available, and 23 patients (14.0%) did not receive a complete RVP. A total of 164 patients were included in the statistical analysis: 30 COVID-19 positive subjects and 134 COVID-19 negative subjects (see Figure). Subject characteristics between the two groups are presented in Table 1.

The rate of SBI in the COVID-19 negative group was 17.9% (95% confidence interval [CI]: 11.8–25.5%) compared to 0% (95% CI: 0.0%-11.1%) in the COVID-19 positive group. Of the SBI in the COVID-19 negative group, 21 subjects had UTI, five had bacteremia, and two had meningitis. For the IBI, three cases of bacteremia were associated with a concurrent UTI, and one case of meningitis was associated with bacteremia and UTI. There was a statistically significant lower rate for any SBI, and specifically for UTI, in the COVID-19 positive group compared to the COVID-19 negative group. When evaluating those with IBI, we found no statistical significance between the COVID-19 positive and COVID-19 negative groups. The results are presented in Table 2. When we performed the subgroup analysis with the infants 29–60 days-old, we found a total of 80 COVID-19 negative patients with a total of 11 SBI. There were 20 COVID-19 positive patients, none of whom had SBI. When the two groups were compared, we found no statistical difference (*P* > 0.05) for any SBI, each of the individual SBI, and IBI. The results are presented in Table 3.

For the age-matched analysis, a total of 30 COVID-19 negative patients were matched to the 30 COVID-19 positive patients. There were zero SBI found in the COVID-19 positive group and a total of six SBI in the COVID-19 negative group. The number of individual SBI was six UTI, one bacteremia, and zero meningitis. Comparing these two groups, the *P*-value was less than 0.05 between all groups except for meningitis as there were no cases in either group (see Table 2).

The last analysis compared SBI rates between patients with any viral infection on RVP vs those with a negative RVP. A total of 21 SBI were found in those with a negative RVP and three SBI in those with concurrent viral infection. When comparing the positive RVP group to the RVP negative, there was a statistically significant lower rate of SBI in the positive RVP group. When looking at the specific SBI, there remained only a statistically significant lower rate of UTI in the positive RVP group compared to the negative RVP group. Of note, there were also seven cases of viral meningitis, which were not included in or calculated as part of the patients with meningitis as this investigation was looking exclusively at IBI. All the patients who tested positive for viral meningitis had a negative bacterial culture and were COVID-19 negative.

## DISCUSSION

The COVID-19 pandemic challenged us with a new disease as well as how to risk-stratify a COVID-19 positive, febrile infant less than 60 days old. Infants less than 60 days old are a vulnerable population for SBI and often need invasive evaluation and treatments. There has been progress in risk-stratifying these infants to avoid more invasive and unnecessary testing. A concurrent viral infection such as RSV has been shown to place febrile infants at a lower risk for SBI.^11^ In this study we aimed to determine whether an infection with SARS-CoV-2 could be used to risk-stratify our patients.

Our results are promising and in line with other published studies that have attempted to address this question. There were zero SBI in any of the COVID-19 positive subjects, and the rate of SBI in the COVID-19 negative subjects is consistent with the rate of SBI in the United States. Moreover, we were able to show a statistically significant difference in any SBI infection between the COVID-19 negative group and COVID-19 positive group. This was in large measure driven by the rate of UTI. The incidence of bacteremia and meningitis has fallen significantly in the US. While the low rate of IBI in the population makes it difficult to obtain findings of statistical significance, we were able to complete a matched analysis that demonstrated a statistically significant lower rate for any SBI and a statistically significant lower rate of UTI and bacteremia in the COVID-19 positive group.

The overall rate of SBI we found in this investigation is similar to the findings in previously published data.^5^,^6^ Moreover, the childhood vaccination rate for Brooklyn’s school district (Kings County) is 94.5%, a sufficiently high level for herd immunity.^13^ The organisms isolated for UTI were *Escherichia coli*, *Enterococcus faecalis*, and *Klebsiella pneumoniae*, while in bacteremia and meningitis the organisms were *Escherichia coli* and Group B *Streptococcus*. These findings are parallel to the current published data.^5^,^14^ The significance of the seven cases of viral meningitis remains unclear. These were COVID-19 negative patients whose bacterial cultures were all negative. New diagnostic testing with PCR allows earlier detection of certain viral pathogens in CSF, which could lead to shorter courses of antibiotics and hospitalizations.

Infants 29–60 days old are of importance as risk stratification based on preliminary laboratory data and urinalysis determines whether more invasive testing with a lumbar puncture is indicated.^7^ Unfortunately, in our investigation, when evaluating this specific age group, we were unable to determine whether SARS-CoV-2 infection affects the subject’s probability of SBI, due to the small sample size.

The AAP’s recently updated guidelines enhance our ability to risk stratify these infants. These guidelines created additional age-group classifications and include specific laboratory studies for inflammatory markers to consider, such as absolute neutrophil count, procalcitonin, and C-reactive protein.^8^ Of note, neither these guidelines nor the PECARN febrile infant guidelines include viral panel results in risk stratification. Respiratory viral panels are becoming more accurate and readily available. With this new information, we must determine how it can be used for the benefit of these vulnerable patients. The use of RVPs will enable us to minimize invasive testing and the use of broad spectrum antibiotics, as well as unnecessary hospitalizations.

## LIMITATIONS

There are several limitations that exist with this study. Retrospective studies are not as strong as prospective studies. However, in this global pandemic with the development of a new illness, it is important to share what is known. It was performed at a single study site, which has inherent limitations including smaller sample size, and which led in turn to an extension of our initial time frame to include more patients. Moreover, our sample size was further reduced due to lower pediatric ED volumes since the beginning of the pandemic. The small sample size limited our ability to describe statistical significance when it comes to bacteremia or bacterial meningitis. Nonetheless, we argue that lack of any bacteremia or bacterial meningitis in the COVID-19 positive cohort is clinically significant.

Lastly, our institution does not routinely screen for invasive intestinal illness given the low prevalence in our community, which may have resulted in missed SBI. Our study highlights the need for additional work to further evaluate and better risk stratify these vulnerable patients, particularly in the context of concurrent viral illnesses. Furthermore, the understanding of the effect of SARS-CoV-2 continues to evolve. The impact that it has had on the pediatric patient continues to increase as new information comes to light.

## CONCLUSION

This investigation contributes to our understanding of the impact of concurrent viral infections on the rate of serious bacterial infection in febrile infants. As advancements in medicine and research evolve, we will improve upon risk-stratification tools for this vulnerable population. Despite the low incidence of SBI in this population, the mortality and morbidity remain significant. While limitations exist within our results and sample size, we believe our study provides a framework to continue the discussion and drive future research on this topic. Several challenges remain in the creation of clear guidelines to risk stratify febrile infants less than 60 days old. We hope that each investigation will contribute to an ever-growing body of knowledge that will establish clear, evidence-based practice for these clinically challenging patients.

## Figures and Tables

**Figure:**
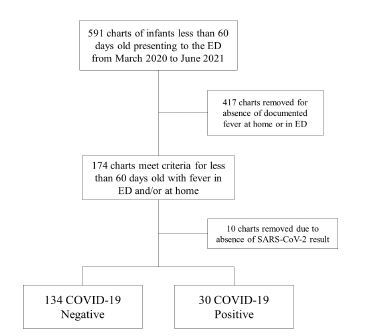
Flow diagram showing the selection of patients for inclusion in study.

**Table 1:** Summary of study participants’ characteristics by group.

	COVID-19 negative n = 134	COVID-19 positive n = 30
Age in days	34 (21 – 44)	34 (25 – 49)
Gender		
Female	53 (39.6%)	15 (50%)
Male		
Race		
White	81 (60.4%)	15 (50%)
Non-White	96 (71.6%)	20 (66.7%)
Ethnicity	38 (28.4%)	10 (33.3%)
Not Hispanic or Latino	114 (85.1%)	26 (86.7%)
Hispanic or Latino	6 (4.5%)	2 (6.7%)
Unknown	14 (10.4%)	2 (6.7%)
Maximum temperature (Celsius)	38.3 (38.1 – 38.8)	38.3 (38.1 – 38.6)
White blood cell count (x10^3^/UL)	12.6 (8.7 – 15.2)	8.4 (6.5 – 11.7)
Absolute neutrophil count (x10^3^/UL)	4.13 (2.50 – 6.80)	2.60 (1.60 – 4.40)
Procalcitonin (ng/mL)	0.65 (0.4 – 1)	0.65 (0.45 – 1.25)

Note: All numeric variables summarized with median and 25th–75th percentile. All categorical variables summarized with frequency and percentage.

*UL*, units per liter; *ng/m*L, nanograms per milliliter.

**Table 2:** Results from unmatched and matched data analyses.

	COVID-19 negative (n = 134)	COVID-19 positive (n = 30)	P-value	Matched Data		

				COVID-19 negative (n = 30)	COVID-19 positive (n = 30)	P-value
Any SBI	24	0	0.008	6	0	<0.001
Bacteremia	5	0	0.59	1	0	<0.001
UTI	21	0	0.02	6	0	<0.001
Bacterial meningitis	2	0	1.00	0	0	n/a
Invasive bacterial infection	6	0	0.59	1	0	<0.001

Note: The variables are summarized with each count. For the unmatched data, the groups were compared using Fisher’s exact test. For the matched data, the groups were compared using McNemar’s test.

*SBI*, serious bacterial infection; *UTI*, urinary tract infection.

**Table 3:** Results from subgroup analysis of 29–60 days old.

	COVID-19 negative (n = 80)	COVID-19 positive (n = 20)	P-value
Any serious bacterial infection	11	0	0.11
Bacteremia	1	0	1.00
Urinary tracty infection	11	0	0.11
Bacterial meningitis	0	0	n/a
Invasive bacterial infection	1	0	1.00

Note: The variables are summarized with each count. The groups were compared using Fisher’s exact test.
